# The Selective Maintenance of Allelic Variation Under Generalized Dominance

**DOI:** 10.1534/g3.116.028076

**Published:** 2016-09-21

**Authors:** Hamish G. Spencer, Cuilodair Mitchell

**Affiliations:** Allan Wilson Centre, Department of Zoology, University of Otago, Dunedin 9054, New Zealand

**Keywords:** multiple alleles, natural selection, mathematical model, polymorphism, selection theory

## Abstract

Simple models of viability selection acting on variation at a single diploid locus only maintain multiple alleles for very restricted sets of fitnesses. Most of these models assume that fitnesses are independent, even if the genotypes share alleles. Here, we extend this result to a model with generalized dominance interactions, in which fitnesses are strongly affected by what we call the “primary effects” of the genotype’s component alleles, so that genotypes with shared alleles have correlated fitnesses. Nevertheless, in keeping with previously reported results, we also show that such fitness sets are easily constructed over time if recurrent mutation is occurring simultaneously. We find that such models maintain less variation over time than do (previous) models with independently sampled fitnesses, especially when the effects of genetic drift are taken into account. We also show that there is a weak tendency for greater weighting of primary effects to evolve over time.

The way in which natural selection molds genetic variation in natural populations has been a central area of investigation since the founding of the field of population genetics (Lewontin 1974; Leffler *et al.* 2012). Of course, variation at different loci will be subject to different population-genetic processes, or combinations thereof, but there is still no unanimity about the relative importance of selection in shaping different parts of the genome role of selection (Mitchell-Olds *et al.* 2007; Leffler *et al.* 2012; Delph and Kelly 2014). Most centrally, does selection actively maintain much of the standing variation observed in natural population’s (the selectionist view; Hahn 2008), or is it largely confined to purifying the population of deleterious mutations (the neutralist view; Kimura 1983)?

Several investigations of the selectionist view have revealed a number of theoretical weaknesses. Most prominently, computer simulations of numerous models of selection showed that only extremely unusual sets of fitnesses were able to maintain more than three or four alleles at a stable equilibrium (Gillespie 1977; Lewontin *et al.* 1978; Clark and Feldman 1986; Marks and Ptak 2001; Trotter and Spencer 2007; Star *et al.* 2007a; Spencer and Chiew 2015). Yet some loci have much larger numbers of alleles. For example, two widely separated populations of *Drosophila pseudoobscura* were found to have ≥ 33 and 22 alleles at the esterase-5 locus (Keith 1983), and 12 and 15 alleles, respectively, at the xanthine dehydrogenase locus (Keith *et al.* 1985). Polymorphism levels revealed in studies such as these were interpreted as being inconsistent with their selective maintenance (Keith 1983; Keith *et al.* 1985).

This feature of selection is not as problematic as it first appears, however. Subsequent studies showed that such unusual fitness sets arose easily when the process of recurrent mutation was incorporated into simulations of selection run over many generations (Spencer and Marks 1988, 1992; Marks and Spencer 1991; Marks and Ptak 2001; Star *et al.* 2007b, 2008; Trotter and Spencer 2008, 2013; Spencer and Chiew 2015). These sorts of models are paralleled by long-term experimental-evolution studies (*e.g.*, Tenaillon *et al.* 2016), but they do not solve the problem completely. For example, Spencer and Marks (1993) pointed out that the models predict too much polymorphism, rarely, if ever, generating a monomorphism. In addition, the distributions of mutational fitnesses used in the simulations were often unrealistic (*e.g.*, uniform on [0,1]).

In this paper, we again model recurrent mutation and selection at a single diploid locus, and examine the patterns of genetic variation produced by these models. But, unlike previous work in which the fitnesses of genotypes containing a newly arising mutation were independent (*e.g.*, Lewontin *et al.* 1978; Spencer and Marks 1988; Marks and Spencer 1991), we constrain these fitnesses to be functions of the individual alleles comprising the genotype plus some, usually lesser weighted, interaction term. In using this form of generalized dominance, we are motivated by the fact that gene products often derive from the coding of just one chromosome. Hence, the fitness of *A_i_A_j_* types in the population may be the average of the fitness contribution of each allele (or some value close to this). Moreover, our formulation means that genotypes that share alleles have correlated, not independent, fitnesses. Our approach can be seen, therefore, as an attempt to improve the realism of mutational distributions in these sorts of investigations.

## Methods

We modeled selection following the standard model of viability selection at a single diploid locus in a large, randomly mating dioecious population (see, *e.g.*, Bürger 2000). Suppose there are *n* alleles, *A*_1_, *A*_2_, …, *A_n_*, at respective frequencies *p*_1_, *p*_2_, …, *p_n_* (with the constraint that $\sum_{i=1}^{n}p_{i}=1$). Then the recurrence equations for the allele frequencies in the next generation are given by

$$p_{i}^{\prime }=\frac{p_{i}}{\bar{w}}\sum_{j=1}^{n}w_{i,j}p_{j}$$ (1)

where *w_i_*_,_*_j_* is the fitness of the *A_i_A_j_* types, and $\bar{w}=\sum_{i=1}^{n}p_{i}\sum_{j=1}^{n}p_{j}w_{i,j}$ is the mean fitness of the population.

We used two different approaches to examine the ability of selection to maintain variation. First, in what we call the “parameter-space approach,” we calculated the *potential* for polymorphism by estimating the proportion of parameter space that maintained all *n* alleles for fixed values of *n* = 1, 2, …5. This approach follows that pioneered by Lewontin *et al.* (1978). Second, following “constructionist approach” of Spencer and Marks (1988), we estimated the likelihood that a system governed by (1), and subject to recurrent mutation, would iterate to parts of parameter space that maintained variation.

We wrote computer simulations (see Supplemental Material, File S1, File S2, File S3, and File S4) that implemented this form of selection under these two approaches. For the parameter-space approach, for a fixed value of *n* we generated *n* primary effects, *X_i_* (*i* = 1, 2, …, *n*) one for each allele, each independently drawn from the uniform distribution on [0,1]. We also sampled $\left(\begin{array}{c} n\\ 2 \end{array}\right)$ interaction effects, *Y_i_*_,_*_j_* (*i* = 1, 2, …, *n*; *j* = *i* + 1, *i* + 2, …, *n*) from the same distribution. The fitness of genotype *A_i_A_j_* was given by the weighted sum

$$w_{i,j}=\alpha \left(X_{i}+X_{j}\right)+\left(1-2\alpha \right)Y_{i,j}$$ (2)

in which the weight, *α*, was a fixed constant between 0 and ½. When *α* = 0, all *w_i_*_,_*_j_* are independent, as in the simulations described in Lewontin *et al.* (1978); when *α* = ½ the fitness of a genotype is simply the average of its component primary effects, and the correlation between *w_i,j_* and *w_i,k_* can be shown to be ½. In general, the correlation between the fitnesses of types sharing one allele is given by

$$Cor\left(w_{i,j},w_{i,k}\right)=\frac{\alpha^{2}}{1-4\alpha +6\alpha^{2}}$$ (3)

Note that when *α* = ½, pairwise heterozygote advantage (*i.e.*, *w_i_*_,_*_j_* > *w_i_*_,_*_i_*, *w_j_*_,_*_j_*) cannot arise and, in general, larger values of *α* reduce the likelihood of this pattern.

We estimated the potential for polymorphism by generating 10^5^ sets of random fitnesses for *n* = 1, 2, … 5. For each fitness set we generated a random initial allele-frequency vector, $\tilde{p}=\left(p_{1},p_{2},\cdots ,p_{n}\right),$ using the broken stick method (Holst 1980). Since any *n*-allele polymorphism is also globally stable (Bürger 2000), the system will iterate to this equilibrium should it exist. In our simulations, an equilibrium was considered to have been reached if $\sum_{i=1}^{n}\left|p_{i}^{\prime }-p_{i}\right|<10^{-8}.$ Otherwise, the system will eventually lose an allele. If, in the simulation, an allele had a frequency of <10^−5^, it was considered to have become extinct, and the iterations were terminated. The potential for polymorphism was calculated as the fraction of fitness sets that afforded a fully polymorphic equilibrium.

For the constructionist approach, we iterated the system for 10^4^ generations, starting with a single allele, *A*_1_, at a frequency of 1.0. Each generation a novel mutation, *A_n_*_+1_, arose at a frequency of 10^−4^. One randomly chosen allele already in the population, selected in proportion to its frequency, simultaneously had its frequency decremented by the same amount. The fitness of *A*_1_*A*_1_ was set to ½; the fitnesses of the newly possible genotypes, *A_i_A_n_*_+1_, were given by Equation (2). When *α* = 0, all *w_i_*_,_*_j_* are independent, as in the simulations described in Spencer and Marks (1988) and Marks and Spencer (1991). Additionally, in one set of simulations, *α* in Equation (2) was replaced by *α_i_*_,_*_n_*_+1_, which was a randomly sampled value from the uniform distribution on [0, ½]. Any allele falling below a frequency of 10^−4^ was considered to have become extinct, and was removed from the simulations. For each set of parameters, we ran 2000 replicate simulations, differing only in the seed for the pseudorandom number generator.

In all simulations we used the algorithm of Marsaglia *et al.* (1990) to generate our pseudorandom numbers.

Our initial simulations ignored the effects of genetic drift. We subsequently incorporated genetic drift, examining the effects of different population sizes, *N*. In these simulations, initial mutational frequencies, and the extinction thresholds in these simulations, were set at 1/(2*N*). Drift took place after selection, before the check for extinct alleles was carried out, and was implemented as a multinomial sampling process with the postselection allele frequencies being the respective multinomial probabilities of sampling each allele.

### Data availability

The authors state that all data necessary for confirming the conclusions presented in the article are represented fully within the article.

## Results

### Parameter-space approach

**Figure 1:**
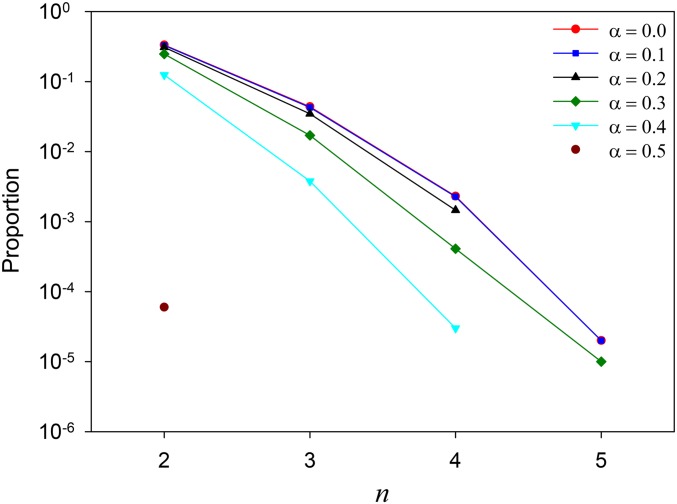
Parameter/state space maintaining all *n* alleles. The red line with filled circles shows the potential for polymorphism with *α* = 0 (as in Lewontin *et al.* 1978); *α* = 0.1: dark blue line with squares; *α* = 0.2: black line with upright triangles. *α* = 0.3: green line with diamonds. *α* = 0.4: light blue line with upside-down triangles. *α* = 0.5: purple circle.

Figure 1 shows the proportion of parameter space that affords a fully polymorphic (*n*-allele) for different values of *α*. Values not plotted mean the proportion is zero (which cannot be shown on the log-scale of the *y* axis). The overall pattern is the same as in all previous investigations (Gillespie 1977; Lewontin *et al.* 1978; Clark and Feldman 1986; Marks and Ptak 2001; Trotter and Spencer 2007; Star *et al.* 2007a; Spencer and Chiew 2015): as *n* increases, the potential for polymorphism declines precipitously.

The results for *α* = 0 are very similar to those of Lewontin *et al.* (1978), as expected. Perhaps surprisingly, there is very little difference between a weighting of *α* = 0 and *α* = 0.1. Larger values of *α* have lower potentials for all values of *n*.

When *α* = 0.5, the fitness of each heterozygote is the mean of the fitnesses of the two corresponding homozygotes. Such additive fitnesses cannot maintain any genetic variation, because the homozygote for one allele always has the largest fitness and that allele will fix. The simulations show that only *n* = 2 gives a nonzero value; 6 (of 10^5^) runs reached an apparent equilibrium before extinction, a consequence of *X*_1_ and *X*_2_ being very close, which results in very weak selection differentials. This nonzero value is thus an artifact.

### Constructionist approach without drift

**Figure 2:**
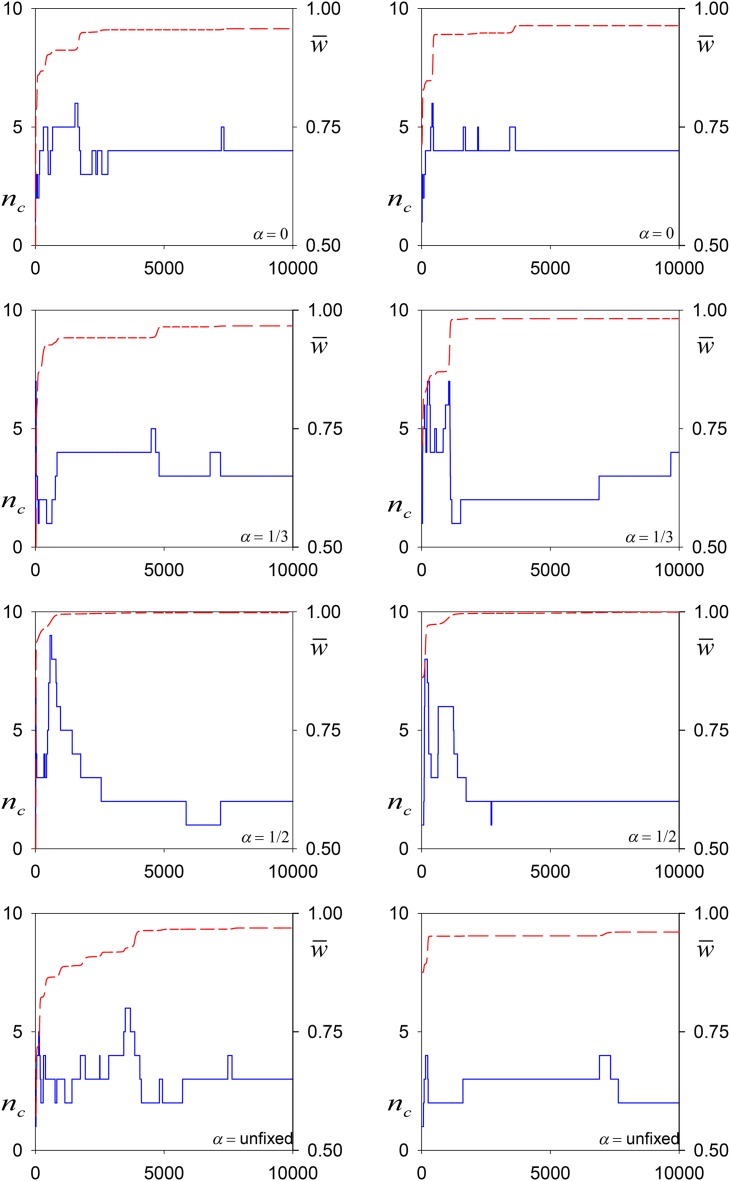
Representative simulations of the construction of polymorphism maintained by viability selection with allelic effects for *α* = 0.0, ⅓, ½, and for when it was unfixed. Upper (dashed red line) shows the mean fitness of the population, $\bar{w},$ and the lower (solid blue) line shows the numbers of common alleles over generations 0–10,000.

In most simulations, especially those with larger values of *α*, many alleles destined for extinction took many generations to fall below the extinction threshold frequency (10^−4^). Such doomed and rare alleles cannot be said to contribute to polymorphism maintained by selection. Moreover, the number of alleles was very sensitive to the value of the extinction threshold, with a smaller value leading to significantly more alleles—always very rare—being present at the end of the simulation. Consequently, we focused on what we called “common alleles,” those at frequencies >0.01. Figure 2 shows the trajectory of the number of common alleles, *n_c_*, and the population mean fitness, $\bar{w},$ for a pair of replicate simulations for three fixed values of *α* (*α* = 0, ⅓, ½) and a further pair for the randomly sampled *α_i_*_,_*_n_*_+1_. In all simulations, *n_c_* increases rapidly at the start (especially for *α* = ½), before a period of frequent invasion by successful mutations and the driving out of existing alleles. Such allelic turnover slows down as time progresses. The mean fitness also increases rapidly at the start of each simulation, and levels off over time.

**Figure 3:**
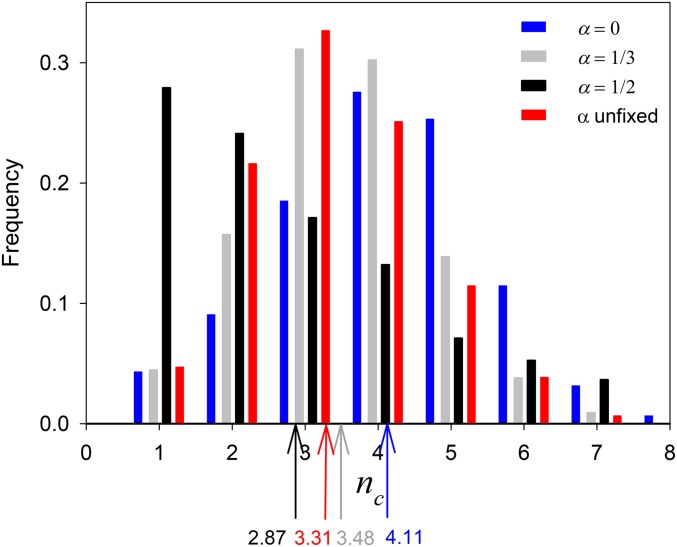
Bar chart of the number of common alleles at Generation 10^4^ in 2000 replicate simulations of the constructionist simulations of viability selection for *α* = 0 (as in Marks and Spencer 1991), ⅓, ½ and for when *α* is unfixed. The respective means are shown by the vertical colored arrows.

Figure 3 shows the distribution of the final number of the common alleles (*i.e.*, after 10^4^ generations), *n_c_*, over the 2000 replicate simulations. We can see that larger *α* values lead to fewer common alleles. Indeed, for *α* = ½, which should not afford any polymorphism at equilibrium, we see that *n_c_* = 1 is the most frequent outcome. Nevertheless, the nonzero frequencies for *n_c_* > 1 demonstrate that these simulations are not at equilibrium at generation 10^4^.

**Figure 4:**
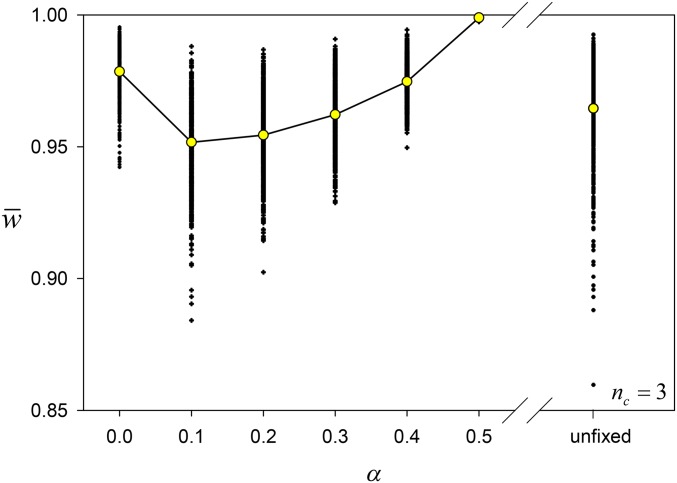
Scatter plot of the values of $\bar{w}$ at Generation 10^4^ in simulations in which the final number of common alleles, *n_c_*, was 3, for different values of *α*. Also shown for the sake of comparison is the spread when *α* was unfixed. Mean values are plotted as yellow circles.

Figure 4 shows, for *n_c_* = 3, the final $\bar{w}$ values of populations and their means as a function of *α*. When *α* = 0, these values are close to the fitness of the fittest homozygote, $\underset{i}{\max}\left(X_{i}\right),$ which is likely to be close to 1.0. Otherwise, the mean fitnesses generally increase from *α* = 0.1 to *α* = 0.5. The mean final value of $\bar{w}$ for simulations with the randomly sampled *α* is 0.96, which is close to the mean value for *α* = 0.3, although the variance is greater. We noted too that the population means of heterozygote fitnesses were always greater than those of homozygote fitnesses; a form of heterozygote advantage evolved.

**Figure 5:**
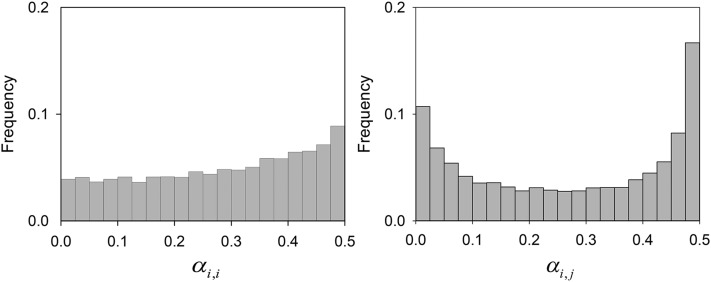
Histogram of the *α* values generated for homozygotes (*α_i,i_*, left) and heterozygotes (*α_i,j_*, right) of common alleles at Generation 10^4^ from 2000 runs of the constructionist simulation when *α* was randomly sampled and free to evolve.

When the values of *α* were randomly sampled, the modal value for *n_c_* was 3, the same as that for *α* = ⅓. The mean value for *n_c_* falls between the means for *α*= ⅓ and *α* = ½. The distribution of the *α_i_*_,_*_i_* and *α_i_*_,_*_j_* (with *i* ≠ *j*) values for common *A_i_* and *A_j_* is shown in Figure 5; the respective means are 0.284 and 0.268. Both distributions have a mode for the largest values, but that for *α_i_*_,_*_j_* is bimodal.

**Figure 6:**
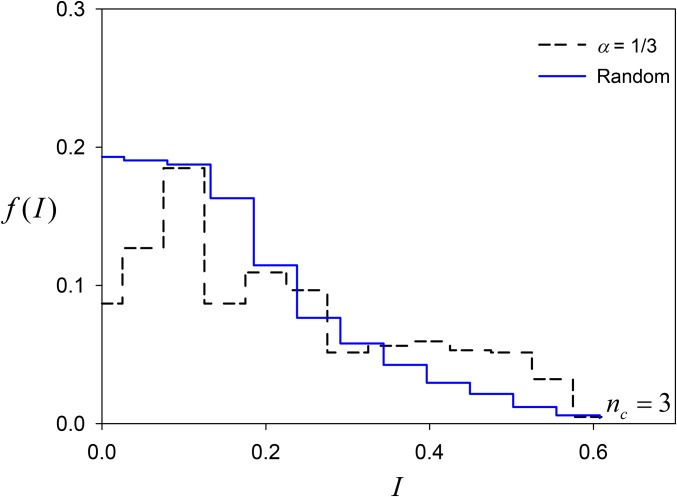
Histograms of $I={\sum_{i=1}^{n_{c}}\left(p_{i}-\frac{1}{n_{c}}\right)}^{2},$ a measure of centrality of common allele frequencies at Generation 10^4^ in the constructionist approach with allelic effects at *α* = ⅓ (*i.e.*, equal parts *X_i_*, *X_j_*, and *Y_i,j_*) for simulations that had three common alleles at Generation 10^4^ (black dashed lines). Also shown (solid blue line) is the distribution expected if allele frequencies were random, values generated using the broken-stick approach.

We also examined the position of the final common-allele-frequency vector, comparing it to that expected if this vector consisted of common-allele frequencies that were randomly uniformly distributed (generated by the broken stick approach). The distribution of values of a measure of centrality,

$$I={\sum_{i=1}^{n_{c}}\left(p_{i}-\frac{1}{n_{c}}\right)}^{2}$$ (4)

where only common alleles are counted, is shown for *n_c_* = 3 and *α* = ⅓ in Figure 6. Smaller values of *I* correspond to more central, even allele frequencies. Simulated polymorphisms were less central than random allele frequencies.

### Constructionist approach with drift

**Figure 7:**
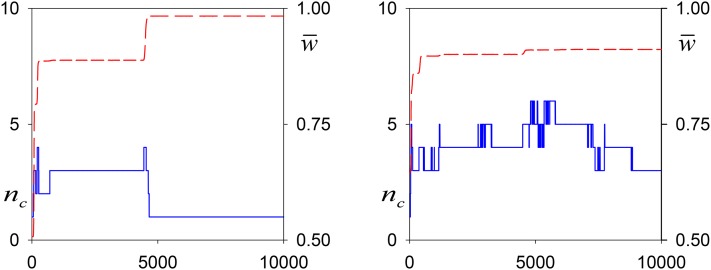
Representative simulations of the construction of polymorphism maintained by viability selection with allelic effects (*α* = ⅓) and drift (*N* = 10^4^). Upper (dashed red line) shows the mean fitness of the population, $\bar{w},$ and the lower (solid blue) line shows the numbers of common alleles over generations 0–10,000.

Figure 7 plots the values of *n_c_* and $\bar{w}$ over time in two typical runs with *N* = 10^4^ and *α* = ⅓. As in the simulations without drift, increases in $\bar{w}$ often correspond to changes—either increases or decreases—in *n_c_*. Nevertheless, one of these simulations shows a remarkable degree of allelic turnover without much change in $\bar{w}.$

**Figure 8:**
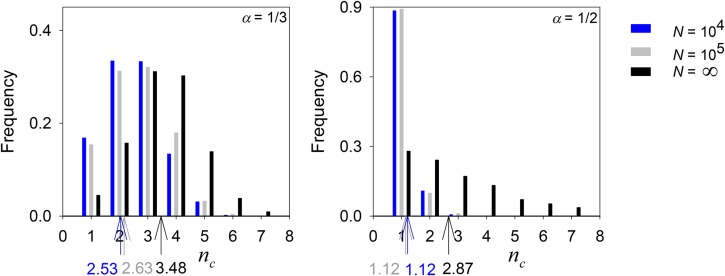
Bar chart comparing the final number of common alleles for *α* = ½, ⅓ for 2000 replicates of the constructionist simulation incorporating genetic drift, with population size, *N* = 10^4^, 10^5^, and ∞ (*i.e.*, no drift), colored blue, gray and black respectively.

Figure 8 shows the distribution from 2000 simulations of *n_c_* after 10^4^ generations for two values of *α* and two of *N*, as well as simulations without drift (effectively *N* = ∞). Smaller populations, in which drift has a greater effect, were likely to harbor fewer alleles, especially for *α* = ½, when drift was more effective at eliminating rare alleles also being slowly removed by selection.

**Figure 9:**
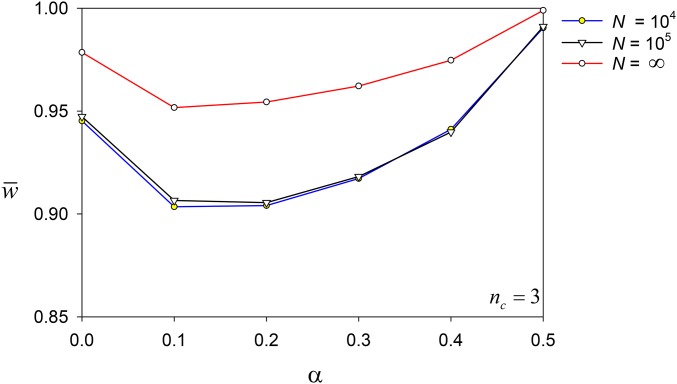
Average $\bar{w}$ at Generation 10^4^ for *N* = 10^4^, 10^5^, and ∞ (blue, black and red lines, respectively) against values of *α*. Only data from simulations with *n_c_* = 3 were used.

Figure 9 shows the means of the final $\bar{w}$ values for different population sizes and values of *α*. The patterns noted above for the simulations without drift are repeated with drift, including the evolution of heterozygote advantage. But drift significantly reduces the means compared to simulations without drift. Even though drift is effective at purging rare deleterious alleles, it also prevents some selectively favored alleles from successfully invading.

**Figure 10:**
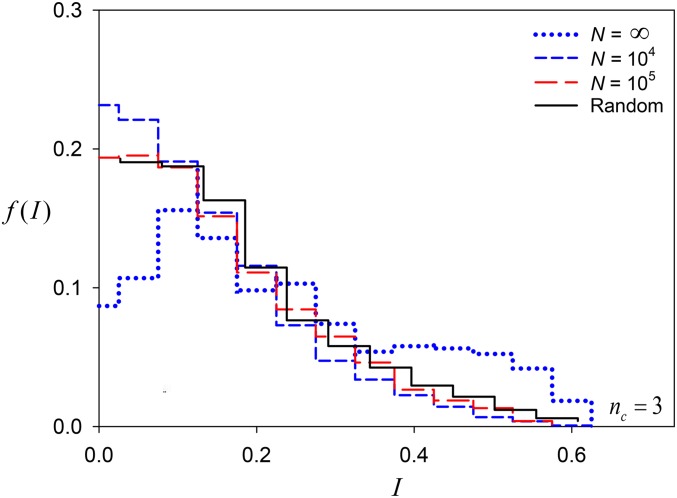
Histograms of $I={\sum_{i=1}^{n_{c}}\left(p_{i}-\frac{1}{n_{c}}\right)}^{2},$ a measure of centrality of common allele frequencies at Generation 10^4^ in the constructionist approach with the allelic effects weighted at *α* = ⅓ for *N* = 10^4^, 10^5^, and ∞ (blue dashed lines, red dashed lines, blue dotted line). Also shown is the distribution expected if allele frequencies were random, values generated using the broken-stick approach (solid black line).

Drift tends to result in more central allele-frequency vectors (Figure 10). Alleles with lower selectively maintained equilibrium frequencies are more likely to be eliminated by drift, resulting in populations with more central allele frequencies.

## Discussion

Our simulations show that constraining genotype fitnesses so that the primary effect of each component allele is heavily weighted (*e.g.*, *α* = 0.4, 0.5) significantly reduces the potential for polymorphism (*i.e.*, the proportion of parameter space affording a fully polymorphic equilibrium) compared to when fitnesses are independent from each other and unconstrained (*α* = 0; the situation in the simulations in Lewontin *et al.* 1978). This difference also holds when a comparison is made between simulations with heavily and weakly weighted (*e.g.*, *α* = 0.1, 0.2) primary effects. Moreover, unlike the case of sex-dependent viabilities (Marks and Ptak 2001) or maternal selection (Spencer and Chiew 2015), this reduced potential is realized when selection and mutation are allowed to construct polymorphisms over time: levels of genetic variation in simulations with more heavily weighted primary effects (*α* = ⅓, ½) are lower compared to when fitnesses are independent from each other and unconstrained (*α* = 0).

Thus, if heavily weighted primary effects underlying our model of generalized dominance are common in nature, heterozygote advantage will be less likely to arise, and the ability of viability selection to maintain variation is likely to be less than previous work has suggested. This finding may answer the objection of Spencer and Marks (1993) that their simulations were too good at generating heterozygote advantage, with consequent high levels of polymorphism. Nevertheless, using a completely different model, namely Fisher’s geometric model of adaptation, Sellis *et al.* (2011) showed that heterozygote advantage should arise commonly, and be an important feature of adaptation, a finding that was confirmed by subsequent experiments with yeast (Sellis *et al.* 2016). Hence, the apparent rarity of good examples of heterozygote advantage in nature remains paradoxical.

Nevertheless, it is clear that, even with heavily weighted primary effects, some alleles destined for a selectively mediated extinction can survive at low frequencies for many generations, provided population sizes are very large. When population size is smaller, genetic drift and selection interact to remove these alleles far more efficiently. This pattern is reminiscent of the way in which drift and selection work together to eliminate deleterious recessive alleles in the standard model of constant viability selection. Drift also significantly reduces the mean fitnesses of populations in our simulations. This difference is likely due to drift eliminating favorable novel mutations at initial low frequencies.

Our simulations showed that, when the weighting of primary effects was allowed to evolve, it did not change greatly. Values for homozygotes, *α_i,i_*, remained almost uniform, with a slight excess of larger values (*α_i,i_* > 0.45). Values for heterozygotes, *α_i,j_*, evolved to become more extreme (compared to the uniform distribution from which they were drawn), with an excess of small (*α_i,j_* < 0.05) and large (*α_i,j_* > 0.45) values. Nevertheless, these results do imply that heavier weighting of primary effects is weakly selected for, which, in turn, is likely to lead to lower levels of polymorphism.

The correspondence between the results adumbrated here and those reported previously suggests that many of the overall patterns in our findings are robust to assumptions about the distribution of mutational fitnesses and the mode of selection. For instance, the potential for maintaining polymorphism plummets with increasing number of alleles for all forms of selection and fitness distributions. Similarly, the constructionist approach shows that this potential grossly underestimates the likelihood that selection and recurrent mutation can generate polymorphism: adding the temporal dimension of recurrent mutation almost always results in more alleles being present in the population than a naïve consideration of the potential might suggest. Nevertheless, better data about the fitness distributions of newly arising mutations would significantly improve the realism of these models.

A second direction in which realism could be improved is to examine the effects of selection at more than one locus. Clearly, selection at one locus has the potential to affect variation at nearby linked loci, including those that are selectively neutral. In an intriguing recent study, Corbett-Detig *et al.* (2015) showed that selective sweeps had a greater effect on levels of linked neutral variation in species with larger populations. Thus, the neutralist expectation of a strong correlation between population size and genetic variation (see Kimura 1983) is counteracted by natural selection acting at linked loci, and the observed correlation is unexpectedly weak. Work in this area is currently underway.

In spite of the rather simplistic assumptions in our models, both in this and previous reports, there is a pleasing match between some of our results and those in recent reports of long-term evolution experiments. For example, in their studies of the evolution of replicated populations of *Escherichia coli* over 50,000 generations, Tenaillon *et al.* (2016) found that most alleles reaching high frequencies were beneficial, and had thus been affected more by selection than drift. They observed, too, that the fraction of beneficial mutations declined as fitness increased, something evident in the long periods of stasis in a number of runs in Figure 2 during which no successful invasions occurred. In our simulations, this result arises because of the fixed upper bound on mutational fitnesses, which means that, as the mean fitness increases, it becomes extremely unlikely that mutations having the capacity to successfully invade will arise. Changing this assumption—for example, by drawing mutational fitnesses from a distribution around the fitness of some parental allele as in Spencer and Marks (1992)—is likely to significantly alter the details and dynamics of the models, although the overall trends as discussed above seem likely to hold. See Spencer and Marks (1992) for a fuller discussion of this issue.

Finally, the ideas outlined above could also be applied to models of frequency-dependent selection. The likelihood of this mode of selection in maintaining polymorphism has long been recognized (Lewontin 1974), and numerous theoretical investigations have generally borne out this heuristic explanation (*e.g.*, Lessard 1984; Asmussen *et al.* 2004; Trotter and Spencer 2007, 2008, 2013; Schneider 2008, 2010).

## Supplementary Material

Supplemental Material
