# Supplementary material for: The Selective Maintenance of Allelic Variation Under Generalized Dominance
Source: G3 (Bethesda). 2016 Sep 21;6(11):3725–32. doi: 10.1534/g3.116.028076 (PMC5100871; doi:10.1534/g3.116.028076)
Supplement: Supplemental Material [file supp_6_11_3725__index.html]

The Selective Maintenance of Allelic Variation Under Generalized Dominance — Supplemental Material 

# The Selective Maintenance of Allelic Variation Under Generalized Dominance

## Supplemental Material for Spencer and Mitchell, 2016

**Files in this Data Supplement:**

- File S1 - Delphi program to implement the parameter-space approach. (.pdf, 47 KB)
- File S2 - Delphi program to implement the constructionist approach without genetic drift for fixed values of α. (.pdf, 47 KB)
- File S3 - Delphi program to implement the constructionist approach without genetic drift for randomly sampled values of α. (.pdf, 49 KB)
- File S4 - Delphi program to implement the constructionist approach with genetic drift for fixed values of α. (.pdf, 60 KB)
