# Supplementary material for: The Selective Maintenance of Allelic Variation Under Generalized Dominance
Source: G3 (Bethesda). 2016 Sep 21;6(11):3725–32. doi: 10.1534/g3.116.028076 (PMC5100871; doi:10.1534/g3.116.028076)
Supplement: Supplemental Material [file supp_g3.116.028076_FileS1.pdf]

```pascal
Program LGT_allelic_effects;
  {$APPTYPE CONSOLE}

 {Classical Constant Viability Selection Model with Weighted Fitness Structure.
  Lewontin, Ginzburg and Tuljapurkar type simulation.}


  uses
  SysUtils;

Const Maxgen = 200000;
        MaxNumAllele = 10;
        MaxNumW = 100000;
        ExtThresh = 0.0001;  {Extinction threshold}
        EquThresh = 0.00000001;  {Equilibrium when sum of absolute Delta
                                    P[I]s < EquThresh}

  Type BigArray = Array[1..MaxNumAllele, 1..MaxNumAllele] of Extended;

  Var IP, JP, Numalleles    :Integer;
      alpha                 :Extended;
      NumW, NumPoly         :Integer;
      Gen                   :0..Maxgen;
      SimpSeed             :Integer; {For Random Number Generation}
      C, CD, CM            :Extended;  {For Random Number Generation}
      Wbar              :Extended;
      Seed              :Array[1..4] of Integer;
      P                 :Array[1..MaxNumAllele] of Extended;
      W                 :BigArray;  {Constants}
      MargW             :Array[1..MaxNumAllele] of Extended;
      U                 :Array[1..97] of Extended;
      Equil, Extinction :Boolean;
      Outdata           :Text;  {Output file for statistical analysis}
      Chkdata           :Text;  {Output file of parameter values}

  Function Uni: Extended;
    {Marsaglia and Zaman's (1990) generator}

    Var Temp    :Extended;

    Begin
    Temp:=U[IP]-U[JP];
    If Temp<0.0 Then Temp:=Temp + 1.0;
    U[IP]:=Temp;
    IP:=IP-1;
    If IP=0 Then IP:=97;
    JP:=JP-1;
    If JP=0 Then JP:=97;
    C:=C-CD;
    If C<0.0 Then C:=C+CM;
    Temp:=Temp-C;
    If Temp<=0.0 Then Uni:=Temp + 1.0 Else Uni:=Temp
    End; {Of Function Uni}

  Procedure Randomize(IR, JR, KR, LR: Integer);
    Var II, JJ, MR    :Integer;
        S, T         :Extended;
    Begin
    For II:=1 To 97 Do
      Begin
      S:=0.0;
      T:=0.5;
      For JJ:=1 To 24 Do
       Begin
        MR:=(((IR*JR) MOD 179)*KR) MOD 179;
        IR:=JR;
       JR:=KR;
       KR:=MR;
       LR:=(53*LR + 1) MOD 169;
       If (LR*MR) MOD 64 >= 32 Then S:=S+T;
       T:=0.5*T
       End;
      U[II]:=S
      End;
```

```
  C:=362436.0/16777216.0;
  CD:=7654321.0/16777216.0;
  CM:=16777213.0/16777216.0;
  IP:=97;
  JP:=33
  End; {Of Procedure Randomize}

Procedure Startup;
  Var Filename      :String;

  Begin
  Writeln;
  Writeln;
  Writeln;
  Writeln('     Lewontin, Ginzburg & Tuljapurkar Type Simulation for');
  Writeln;
  Writeln('Classical Constant Viability Selection Model with Weighted Fitness Structure');
  Writeln;
  Writeln('                             1 Deme');
  Writeln;
  Writeln('    Hamish G. Spencer & Cuilodair Mitchell, November 2015');
  Writeln;
  Writeln;

  {Read in parameter values}
  Write('Enter random number seed: ');
  Readln(SimpSeed);
  Writeln;

  {Prepare Output file}
  Writeln('The output filenames will start with LGTWeight_');
  Write('Enter any further characters required in the name: ');
  Readln(Filename);
  Writeln('Please enter a value for alpha: ');
  Readln(alpha);
  Writeln;
  Filename:='LGTWeight_' + Filename;
  Assign(Outdata, Filename + '.OUT');
  Rewrite(Outdata);

  {Produce Checking file}
  Assign(Chkdata, Filename + '.CHK');
  Rewrite(Chkdata);
  Writeln(Chkdata, 'Program LGT1Weight: Input parameters for file ', Filename, ':');
  Writeln(Chkdata);
  Writeln(Chkdata, 'alpha: ', alpha:5);
  Writeln(Chkdata, 'Number of alleles: ', Numalleles:2);
  Writeln(Chkdata, 'Random  number seed: ', SimpSeed);
  Writeln(Chkdata, 'ExtThresh = ', ExtThresh:20:18);
  Writeln(Chkdata, 'EquThresh = ', EquThresh:20:18);
  Writeln(Chkdata, 'MaxGen = ', Maxgen:6);
  Writeln(Chkdata, 'Number of W matrices = ', MaxNumW:6);
  Close(Chkdata);

  End; {Of Procedure Startup}

Procedure Mutation;  {Haploid Model}

  Var I, J  :Integer;
      X     :Array[1..MaxNumAllele] of Extended;

  Begin
  For I:= 1 To NumAlleles Do X[I] := Uni;
  For I:=1 To NumAlleles Do For J:=I To NumAlleles Do
    Begin
    W[I, J]:= alpha*(X[I]+X[J]) + (1.0 - 2.0*alpha)*Uni;
    W[J, I]:=W[I, J]
    End
  End; {Of Procedure Mutation}

Procedure DeltaP;
  {Performs the changes in allele frequencies.}

  Var I, J                :Integer;
```

```
    TempMarg, OldP, SumDP   :Extended;

  Begin
  SumDP:=0.0;
  {First, calculate new marginal viabilities}
  For I:=1 to Numalleles Do
    Begin
    TempMarg:=0.0;
    For J:=1 To Numalleles Do TempMarg:=TempMarg + P[J]*W[I, J];
    MargW[I]:=TempMarg
    End;

  {Calculate new Wbar}
  Wbar:=0.0;
  For I:=1 To Numalleles Do Wbar:=Wbar + P[I]*MargW[I];

  {Calculate new P[I]s and add up absolute changes}
  For I:=1 To Numalleles Do
    Begin
    OldP:=P[I];
    P[I]:=P[I]*MargW[I]/Wbar;
    SumDP:=SumDP + Abs(OldP - P[I]);
    Extinction := Extinction Or (P[I] < ExtThresh)
    End;

  {Check for equilibrium}
  Equil:=SumDP<EquThresh;

  End; {Of Procedure DeltaP}

Procedure OneP;
  {Generates a random P vector, using the broken stick method, and
   then iterates this vector until either equilibrium is reached
   or the extinction of one allele occurs.}

  Type Glsarray = Array[1..MaxNumAllele-1] of Extended;

  Var I              :Integer;
      Stick          :Glsarray;

  Procedure PikSrt(NS: Integer; Var Arr: Glsarray);
    {From Press et al.: 723}

    Label 10;

    Var IS1, JS   :Integer;
        AS1       :Extended;

    Begin
    For JS:=2 To NS Do
      Begin
      AS1:=Arr[JS];
      For IS1:=JS - 1 DownTo 1 Do
        Begin
        If (Arr[IS1] <= AS1) Then GoTo 10;
        Arr[IS1+1]:=Arr[IS1]
        End;
      IS1:=0;
10:   Arr[IS1+1]:=AS1
      End
    End;  {Of Procedure PikSrt}

  Begin
  {First, generate random P}
  For I:=1 To Numalleles - 1 Do Stick[I]:=Uni;
  PikSrt(Numalleles - 1, Stick);
  P[1]:=Stick[1];
  For I:=2 To Numalleles - 1 Do P[I]:=Stick[I] - Stick[I - 1];
  P[Numalleles]:=1.0 - Stick[Numalleles - 1];

  {Now iterate until equilibrium}
  Gen:=0;
  Equil:=False;
  Repeat
```

```
    Gen:=Gen+1;
    DeltaP
  Until Extinction Or Equil

  End;  {Of Procedure OneP}

Procedure OneW;

  Begin
  {Set up W}
  Mutation;

  Extinction := False;
  OneP {Since we have constant viability selection, just one P is needed to characterise W}

  End; {Of OneW}

Begin {******* Main Program ********}
Startup;
For NumAlleles:=2 To MaxNumAllele Do
  Begin
  Seed[1]:=(SimpSeed + NumAlleles) MOD 178 + 1;
  Seed[2]:=(SimpSeed + NumAlleles + (NumAlleles DIV 178)) MOD 178 + 1;
  Seed[3]:=(SimpSeed + NumAlleles + ((NumAlleles DIV 178 + NumAlleles) DIV 178)) MOD 178 + 1;
  Seed[4]:=(SimpSeed + NumAlleles) MOD 169;
  Randomize(Seed[1], Seed[2], Seed[3], Seed[4]);
  NumPoly := 0;
  For NumW:=1 To MaxNumW Do
    Begin
    OneW;
    If Not Extinction Then NumPoly := NumPoly + 1
    End;
  Writeln(NumAlleles:4, NumPoly/MaxNumW:10:6);
  Writeln(Outdata, NumAlleles:4, NumPoly/MaxNumW:10:6)
  End;
Close(Outdata);
Writeln;
Writeln('Program successfully completed!');
Writeln;
Writeln('Hit any Enter key to continue');
Readln
End. {Of Program LGT1Deme}
```
