# Supplementary material for: The Selective Maintenance of Allelic Variation Under Generalized Dominance
Source: G3 (Bethesda). 2016 Sep 21;6(11):3725–32. doi: 10.1534/g3.116.028076 (PMC5100871; doi:10.1534/g3.116.028076)
Supplement: Supplemental Material [file supp_g3.116.028076_FileS2.pdf]

```
Program SandCM;
  {$APPTYPE CONSOLE)}
  {One Run of Spencer & Marks Simulation, with Correlated Fitness Structure.
   Allelic Effects Xi & Xj, as well as Genotype Effects Yij.}

  uses
  SysUtils,
  Math;

Const Maxgen = 10000;
        Maxallele = 100;
        ExtThresh = 0.0001;   {Extinction threshold}
        MaxRunCount = 1;

  Type BigArray = Array[1..Maxallele, 1..Maxallele] of Extended;

  Var N                     :Integer;
      RunCount              :Integer; {To keep count of the ammount of runs}
      SimpSeed              :Integer;
      alpha                 :Extended; {weighting of allelic and genotypic effects}
      Wbar                  :Extended;
      IP, JP               :Integer; {For Random Number Generation}
      C, CD, CM             :Extended;  {For Random Number Generation}
      Seed               :Array[1..4] of Integer;
      P                  :Array[1..Maxallele] of Extended;
      X                  :Array[1..Maxallele] of Extended;
      W                  :BigArray;  {Constants}
      U                  :Array[1..97] of Extended;
      Outdata            :Text;  {Output file for statistical analysis}


  Function Uni: Extended;
    {Marsaglia et al. (1990) generator}

    Var Temp    :Extended;

    Begin
    Temp:=U[IP]-U[JP];
    If Temp<0.0 Then Temp:=Temp + 1.0;
    U[IP]:=Temp;
    IP:=IP-1;
    If IP=0 Then IP:=97;
    JP:=JP-1;
    If JP=0 Then JP:=97;
    C:=C-CD;
    If C<0.0 Then C:=C+CM;
    Temp:=Temp-C;
    If Temp<=0.0 Then Uni:=Temp + 1.0 Else Uni:=Temp
    End; {Of Function Uni}

  Procedure Randomize(IR, JR, KR, LR: Integer);
    Var II, JJ, MR     :Integer;
        S, T          :Extended;
    Begin
    For II:=1 To 97 Do
      Begin
      S:=0.0;
      T:=0.5;
      For JJ:=1 To 24 Do
       Begin
        MR:=(((IR*JR) MOD 179)*KR) MOD 179;
        IR:=JR;
       JR:=KR;
       KR:=MR;
       LR:=(53*LR + 1) MOD 169;
       If (LR*MR) MOD 64 >= 32 Then S:=S+T;
       T:=0.5*T
       End;
      U[II]:=S
      End;
    C:=362436.0/16777216.0;
    CD:=7654321.0/16777216.0;
    CM:=16777213.0/16777216.0;
    IP:=97;
```

```pascal
  JP:=33
  End; {Of Procedure Randomize}

Procedure Startup;

  Var FileName        :String;


  Begin
  Writeln;
  Writeln;
  Writeln;
  Writeln('                 Spencer & Marks Type Simulation for');
  Writeln;
  Writeln('  Classical Constant Viability Selection Model with Weighted Fitness Structure');
  Writeln;
  Writeln('       Hamish G. Spencer & Cuilodair Mitchell, November 2015');
  Writeln;
  Writeln;

  {Read in parameter values}
  Write('Enter random number seed: ');
  Readln(SimpSeed);
  Writeln;

  Repeat
  Write('Enter a value for the parameter alpha on the range [0, 0.5]: ');
  Readln(alpha)
  Until (alpha >= 0) and (alpha <= 0.50);

  {Prepare Output file}
  Writeln('The output filenames will start with SandCM_ and end with _alpha' + FloatToStr(alpha))
  Write('Enter any further characters required in the name: ');
  Readln(Filename);
  Filename:= 'SandCM_' + Filename + '_alpha_' + FloattoStr(alpha) + '.txt';
  Assign(Outdata, Filename);
  Rewrite(Outdata)

  End; {Of Procedure Startup}

Procedure Mutation;

  Var I, Parent   :Integer;
      ParentThresh, SumFreq    : Extended;

  Begin
  ParentThresh := Uni;
  Parent := 0;
  SumFreq := 0.0;
  Repeat
    Parent := Parent + 1;
    SumFreq := SumFreq + P[Parent]
  Until SumFreq >= ParentThresh;
  {Parent is the existing allele that is going to mutate}
  If P[Parent] < ExtThresh Then
  {It is very rare and we need to ensure we don't get a negative P[N + 1]}
    Begin
    P[N + 1] := P[Parent];
    P[Parent] := 0.0
    End
  Else {P[Parent] >= ExtThresh}
    Begin
    P[N + 1] := ExtThresh;
    P[Parent] := P[Parent] - ExtThresh
    End;
  X[N+1] := Uni;
  For I:= 1 To N Do
    Begin
    {Allelic effects are implemented in this block, on lines 145, 150 and 217.
    Generate fitnesses for all new Wi,n+1 pairs using equation 2. }
    W[I, N+1] := alpha*(X[I]+X[N+1]) + ((1-2*alpha)*Uni);
    {Fitness matrix is symmetrical. Apply symmetry operations}
    W[N+1, I] := W[I, N+1]
    End;
```

```
  {calculate fitness for the new homozygote}
  W[N+1, N+1] := alpha*(2.0*X[N+1]) + ((1-2*alpha)*Uni);
  N := N+1
  End; {Of Procedure Mutation}

Procedure Selection;
  {Performs the changes in allele frequencies.}

  Var I, J, K              :Integer;
      TempMarg             :Extended;
      MargW                :Array[1..Maxallele] of Extended;

  Begin
  {First, calculate new marginal viabilities}
  For I:=1 to N Do
    Begin
    TempMarg:=0.0;
    For J:=1 To N Do TempMarg:=TempMarg + P[J]*W[I, J];
    MargW[I]:=TempMarg
    End;

  {Calculate new Wbar}
  Wbar:=0.0;
  For I:=1 To N Do Wbar:=Wbar + P[I]*MargW[I];

  {Calculate new P[I]s}
  For I:=1 To N Do P[I]:=P[I]*MargW[I]/Wbar;

  {Check for extinct alleles, delete any which have gone extinct and replace
   the position in the fitness matrix with the allele from the last row/col }

  K:=0;
  Repeat
    K:=K+1;
    If P[K] < ExtThresh Then
      Begin
      For I:=1 To N-1 Do
        Begin
        W[I,K]:=W[I,N];
        W[K,I]:=W[N,I]
        End;
      W[K,K] := W[N,N];
      P[K] := P[N];
      X[K] := X[N];
      N := N-1;
      K := K-1
      End
  Until K >= N

  End; {Of Procedure Selection}

{Will do one run of the simulation for MaxGen generations. The total number
  of runs is set by the global variable MaxRunCount }
Procedure OneRun;
var Gen                        :1..MaxGen;
    IR, Common             :Integer;
    I, Ic                      :Extended;
Begin
  Seed[1]:=(SimpSeed + RunCount) MOD 178 + 1;
  Seed[2]:=(SimpSeed + RunCount + (RunCount DIV 178)) MOD 178 + 1;
  Seed[3]:=(SimpSeed + RunCount + ((RunCount DIV 178 + RunCount) DIV 178)) MOD 178 + 1;
  Seed[4]:=(SimpSeed + RunCount) MOD 169;
  Randomize(Seed[1], Seed[2], Seed[3], Seed[4]);

  {Set up Fitness matrix}
  {set uniform random number on [0, 1] to allelic effect of X1}
  X[1] := Uni;
  {Calculate fitness of the first allele}
  W[1,1] := (alpha*2.0*X[1]) + ((1-2*alpha)*Uni);
  N := 1;
  P[1] := 1.0;

  {Simulate one run of MaxGen generations}
  For Gen :=1 To MaxGen Do
```

```
    Begin
    Mutation;
    Selection;
    {Alleles with a frequency greater than 0.01 are considered "common" and are
    counted by the Common integer}
    Common := 0;
    For IR := 1 To N Do If P[IR] > 0.01 Then Common := Common + 1;
    {output each generation}
    Writeln(Outdata, Gen:5, N:5, Common:5, Wbar:10:4);
    End;

  {Output at the end of each run.
  Common := 0;
  Ic := 0;

  For IR := 1 To N Do If P[IR] > 0.01 Then Common := Common + 1;
  //Calculate I for common alleles (Ic)
  For IR := 1 To N Do If P[IR] > 0.01 Then Ic := Ic + Power( (P[IR]- 1/Common), 2);


  Writeln(Outdata, RunCount:5, N:5, Common:5, Ic:5, Wbar:10:4); }

End;

Begin {******* Main Program ********}
Startup;
For RunCount := 1 to MaxRunCount do OneRun;

Close(Outdata);

Writeln;
Writeln;
Writeln('Program successfully completed!');
Writeln;
Writeln('Hit any Enter key to continue');
Readln
End. {Of Program SandCM}
```
