# Supplementary material for: The Selective Maintenance of Allelic Variation Under Generalized Dominance
Source: G3 (Bethesda). 2016 Sep 21;6(11):3725–32. doi: 10.1534/g3.116.028076 (PMC5100871; doi:10.1534/g3.116.028076)
Supplement: Supplemental Material [file supp_g3.116.028076_FileS4.pdf]

```
Program SandCM_drift;
  {$APPTYPE CONSOLE)}
  {One Run of Spencer & Marks Simulation, with Correlated Fitness Structure.
   Allelic Effects Xi & Xj, as well as Genotype Effects Yij.}

  uses
  SysUtils,
  Math;

  Const Maxgen = 10000;
        PopSize = 100000;
        Maxallele = 100;
        ExtThresh = 0.000005;  {Extinction threshold}
        MaxRunCount = 2000;


  Type BigArray = Array[1..Maxallele, 1..Maxallele] of Extended;

  Var N                     :Integer;
      RunCount              :Integer; {To keep count of the ammount of runs}
      SimpSeed              :Integer;
      alpha                 :Extended; {determine which factors determine the
                                        fitness of new allelic combinations.
                                        On the interval [0, 0.5] }
      Wbar                  :Extended;
      IP, JP               :Integer; {For Random Number Generation}
      C, CD, CM             :Extended;  {For Random Number Generation}
      Seed               :Array[1..4] of Integer;
      P                  :Array[1..Maxallele] of Extended;
      X                  :Array[1..Maxallele] of Extended;
      W                  :BigArray;  {Constants}
      U                  :Array[1..97] of Extended;
      Outdata            :Text;  {Output file for statistical analysis}


  Function Uni: Extended;
    {Marsaglia et al. (1990) generator}

    Var Temp    :Extended;

    Begin
    Temp:=U[IP]-U[JP];
    If Temp<0.0 Then Temp:=Temp + 1.0;
    U[IP]:=Temp;
    IP:=IP-1;
    If IP=0 Then IP:=97;
    JP:=JP-1;
    If JP=0 Then JP:=97;
    C:=C-CD;
    If C<0.0 Then C:=C+CM;
    Temp:=Temp-C;
    If Temp<=0.0 Then Uni:=Temp + 1.0 Else Uni:=Temp
    End; {Of Function Uni}

  Procedure Randomize(IR, JR, KR, LR: Integer);
    Var II, JJ, MR     :Integer;
        S, T          :Extended;
    Begin
    For II:=1 To 97 Do
      Begin
      S:=0.0;
      T:=0.5;
      For JJ:=1 To 24 Do
       Begin
        MR:=(((IR*JR) MOD 179)*KR) MOD 179;
        IR:=JR;
       JR:=KR;
       KR:=MR;
       LR:=(53*LR + 1) MOD 169;
       If (LR*MR) MOD 64 >= 32 Then S:=S+T;
       T:=0.5*T
       End;
      U[II]:=S
      End;
```

```
    C:=362436.0/16777216.0;
    CD:=7654321.0/16777216.0;
    CM:=16777213.0/16777216.0;
    IP:=97;
    JP:=33
    End; {Of Procedure Randomize}

  Function GammLn(XX : Extended):Extended;

    Const Stp = 2.50662827465;

    Var Xg, Tmpg, Serg : Extended;
        Jg             : Integer;
        Cofg           :Array[1..6] of Extended;

    Begin
    Cofg[1] := 76.18009173;
    Cofg[2] := -86.50532033;
    Cofg[3] := 24.01409822;
    Cofg[4] := -1.231739516;
    Cofg[5] := 0.120858003E-2;
    Cofg[6] := -0.536382E-5;
    Xg := XX - 1.0;
    Tmpg := Xg + 5.5;
    Tmpg := (Xg + 0.5)*Ln(Tmpg)-Tmpg;
    Serg := 1.0;
    For Jg := 1 To 6 Do
      Begin
      Xg := Xg + 1.0;
      Serg := Serg + Cofg[Jg]/Xg
      End;
    Gammln := Tmpg + Ln(Stp*Serg)
    End; {Of Gammln}

  Function Bin(PP:Extended; NB: Integer): Integer;
           {Algorithm from Press et al}
    Label 1;
    Var Am, Em, En, G, Angle   : Extended;
        OldG, Pb, Pc, Bnl      : Extended;
        PcLog, Plog, Pold, Sq, T, Y  : Extended;
        Jb, Nold                 : Integer;

    Begin
    Nold := -1; Pold := -1.0;
    If (PP <= 0.5) Then Pb := PP Else Pb := 1.0 - PP;
    Am := NB*Pb;
    If (NB < 25) Then
      Begin
      Bnl := 0.0;
      For Jb := 1 To NB Do
        Begin
        If (Uni < Pb) Then Bnl := Bnl + 1.0
        End
      End
    Else
      If (Am < 1.0) Then
        Begin
        G := Exp(-Am); T := 1.0;
        For Jb := 0 To NB Do
          Begin
          T := T*Uni;
          If (T < G) Then GoTo 1
          End;
        Jb := NB;
1:      Bnl := Jb
        End
      Else
        Begin
        If (NB <> Nold) Then
          Begin
          En := NB;
          OldG := GammLn(En + 1.0);
          Nold := NB
          End;
```

```
      If (Pb <> POld) Then
        Begin
        Pc := 1.0 - Pb;
        Plog := Ln(Pb);
        Pclog := Ln(Pc);
        Pold := Pb
        End;
      Sq := Sqrt(2.0*Am*Pc);
      Repeat
        Repeat
          Angle := Pi*Uni;
          Y := Sin(Angle)/Cos(Angle);
          Em := Sq*Y + Am
        Until ((Em >= 0.0) AND (Em < En + 1.0));
        Em := Trunc(Em);
        T := 1.2*Sq*(1.0+Sqr(Y))*Exp(OldG-Gammln(Em+1.0) - Gammln(En-Em+1.0) + Em*PLog + (En-Em)*
      Until (Uni <= T);
      Bnl := Em
      End;
    If (Pb <> PP) Then Bnl := NB - Bnl;
    Bin := Round(Bnl)
    End; {Of Binomial}


  Procedure Startup;

    Var FileName        :String;


    Begin
    Writeln;
    Writeln;
    Writeln;
    Writeln('                Spencer & Marks Type Simulation for');
    Writeln;
    Writeln('  Classical Constant Viability Selection Model with Weighted Fitness Structure');
    Writeln;
    Writeln('     Hamish G. Spencer & Cuilodair Mitchell, November 2015');
    Writeln;
    Writeln;

    {Read in parameter values}
    Write('Enter random number seed: ');
    Readln(SimpSeed);
    Writeln;
    Repeat
    Write('Enter a value for the parameter alpha on the range [0, 0.5]: ');
    Readln(alpha)

    Until (alpha >= 0) and (alpha <= 0.50);


    {Prepare Output file}
    Writeln('The output filenames will start with SandCM_ and end with _alpha' + FloatToStr(alpha))
    Write('Enter any further characters required in the name: ');
    Readln(Filename);
    Filename:= 'SandCM_' + Filename + '_alpha_' + FloattoStr(alpha) + '.txt';
    Assign(Outdata, Filename);
    Rewrite(Outdata)

    End; {Of Procedure Startup}

  Procedure Mutation;

    Var I, Parent  :Integer;
        ParentThresh, SumFreq   : Extended;

    Begin
    ParentThresh := Uni;
    Parent := 0;
    SumFreq := 0.0;

    Repeat
      Parent := Parent + 1;
```

```
      SumFreq := SumFreq + P[Parent]
    Until SumFreq >= ParentThresh;
    {Parent is the existing allele that is going to mutate}
    If P[Parent] < ExtThresh Then
    {It is very rare and we need to ensure we don't get a negative P[N + 1]}
      Begin
      P[N + 1] := P[Parent];
      P[Parent] := 0.0
      End
    Else {P[Parent] >= ExtThresh}
      Begin
      P[N + 1] := ExtThresh;
      P[Parent] := P[Parent] - ExtThresh
      End;
    X[N+1] := Uni;
    For I:= 1 To N Do
      Begin
      {Allelic effects are implemented in this block, on lines 242, 247 and 337.
      Generate fitnesses for all new Wi,n+1 pairs using equation 2. }
      W[I, N+1] := alpha*(X[I]+X[N+1]) + ((1-2*alpha)*Uni);
      {Fitness matrix is symmetrical. Apply symmetry operations}
      W[N+1, I] := W[I, N+1]
      End;
      {calculate fitness for the new homozygote}
    W[N+1, N+1] := alpha*(2.0*X[N+1]) + ((1-2*alpha)*Uni);
    N := N+1
    End; {Of Procedure Mutation}

  Procedure Selection;
    {Performs the changes in allele frequencies.}

    Var I, J                :Integer;
        TempMarg            :Extended;
        MargW               :Array[1..Maxallele] of Extended;

    Begin
    {First, calculate new marginal viabilities}
    For I:=1 to N Do
      Begin
      TempMarg:=0.0;
      For J:=1 To N Do TempMarg:=TempMarg + P[J]*W[I, J];
      MargW[I]:=TempMarg
      End;

    {Calculate new Wbar}
    Wbar:=0.0;
    For I:=1 To N Do Wbar:=Wbar + P[I]*MargW[I];

    {Calculate new P[I]s}
    For I:=1 To N Do P[I]:=P[I]*MargW[I]/Wbar;
    End; {Of Procedure Selection}

  Procedure Drift;

    var I, AllelesLeft        :Integer;
        Pleft        :Extended;
        PopAlleles   :Array[1..MaxAllele] of Integer;

    Begin
    AllelesLeft := PopSize*2;
    Pleft := 1.0;
    For I := 1 To N-1 Do
      Begin
      PopAlleles[I] := Bin(P[I]/Pleft, AllelesLeft);
      AllelesLeft := AllelesLeft - PopAlleles[I];
      Pleft := Pleft - P[I]
      End;
    PopAlleles[N] := AllelesLeft;

    For I := 1 to N Do P[I] := PopAlleles[I] / (2*PopSize)
    End; {Of Procedure Drift}

  Procedure ExtCheck;
    {Check for extinct alleles, delete any which have gone extinct and replace
```

```
   the position in the fitness matrix with the allele from the last row/col }
  Var I, K                 :Integer;

  Begin
  K:=0;
  Repeat
    K:=K+1;
    If P[K] < ExtThresh Then
      Begin
      For I:=1 To N-1 Do
        Begin
        W[I,K]:=W[I,N];
        W[K,I]:=W[N,I]
        End;
      W[K,K] := W[N,N];
      P[K] := P[N];
      X[K] := X[N];
      N := N-1;
      K := K-1
      End
  Until K >= N
  End; {Of Procedure ExtCheck}


{Will do one run of the simulation for MaxGen generations. The total number
  of runs is set by the global variable MaxRunCount }
Procedure OneRun;
var Gen                         :1..MaxGen;
    IR, Common              :Integer;
    Ic                       :Extended;
Begin
  Seed[1]:=(SimpSeed + RunCount) MOD 178 + 1;
  Seed[2]:=(SimpSeed + RunCount + (RunCount DIV 178)) MOD 178 + 1;
  Seed[3]:=(SimpSeed + RunCount + ((RunCount DIV 178 + RunCount) DIV 178)) MOD 178 + 1;
  Seed[4]:=(SimpSeed + RunCount) MOD 169;
  Randomize(Seed[1], Seed[2], Seed[3], Seed[4]);

  {Set up Fitness matrix}
  X[1] := Uni;
  {Calculate fitness of first pair of alleles.}
  W[1,1] := (alpha*2.0*X[1]) + ((1-2*alpha)*Uni);
  N := 1;
  P[1] := 1.0;

  {Simulate one run of MaxGen generations}
  For Gen :=1 To MaxGen Do
    Begin
    Mutation;
    Selection;
    Drift;
    ExtCheck;
    {Alleles with a frequency greater than 0.01 are considered "common" and are
    counted by the Common integer        }
    {Output each generation.
    Common := 0;
    For IR := 1 To N Do If P[IR] > 0.01 Then Common := Common + 1;
    Writeln(Outdata, Gen:5, N:5, Common:5, Wbar:10:4);               }
    End;

  {Ouput at the end of each run.
  Ic := 0;
  Common := 0;
  For IR := 1 To N Do If P[IR] > 0.01 Then Common := Common + 1;

  //calculate the I for all alleles common alleles (Ic)
  For IR := 1 To N Do If P[IR] > 0.01 Then Ic := Ic + Power( (P[IR]- 1/Common), 2);
  Writeln(Outdata, RunCount:5, N:5, Common:5, Wbar:10:4);    }

End;

Begin {******* Main Program ********}
Startup;
For RunCount := 1 To MaxRunCount Do OneRun;
```

```
Close(Outdata);

Writeln;
Writeln;
Writeln('Program successfully completed!');
Writeln;
Writeln('Hit any Enter key to continue');
Readln
End. {Of Program SandCM}
```
